# The Intricate Dance Between Inflammation and Myeloproliferative Neoplasms: From Origins to Outcomes

**DOI:** 10.1007/s11899-026-00774-5

**Published:** 2026-03-06

**Authors:** Angela G Fleischman

**Affiliations:** Division of Hematology/Oncology, University of California, Irvine, CA USA

**Keywords:** Myeloproliferative neoplasm, Inflammation, Immune dysregulation, JAK/STAT signaling

## Abstract

**Purpose of Review:**

Myeloproliferative neoplasms (MPNs) lie at the intersection of malignancy and chronic inflammatory disease. This review summarizes current understanding of how inflammation drives MPN pathogenesis, from clonal initiation to progression and symptom burden, and explores how emerging therapies modulate the inflammatory microenvironment.

**Recent Findings:**

Evidence from human genetics, epidemiology, and experimental models shows that chronic inflammatory stress promotes the expansion of JAK2- and other MPN-associated clones. Inflammatory cytokine networks sustain myeloproliferation, reshape the bone marrow niche, and contribute to fibrosis. JAK inhibitors remain the cornerstone of therapy and exert much of their clinical benefit through suppression of cytokine signaling. Newer agents also mitigate inflammation through complementary mechanisms.

**Summary:**

Inflammation is inseparable from MPN biology and represents both a driver and a therapeutic target. Reframing MPN as a disorder of maladaptive immune and stromal interactions highlights opportunities to restore balance within this ecosystem and potentially alter disease course.

## Introduction

MPNs occupy a unique position at the interface of cancer and chronic inflammatory disease, with dysregulated cytokine production both fueling clonal expansion and shaping the systemic manifestations of the disorder. Inflammation contributes to MPN biology across the disease spectrum, beginning with its role in clonal emergence and disease initiation, extending through its impact on symptom burden, and culminating in its contribution to progression toward myelofibrosis and leukemic transformation. The close relationship between MPN and inflammatory disease is further highlighted by their overlapping treatment strategies: JAK inhibitors, central to MPN therapy, are also widely used to treat autoimmune and autoinflammatory disorders. This review will discuss the involvement of inflammation at each step in the MPN disease process.

### Inflammation as a Driver of Disease Initiation in MPN

An emerging theme in myeloid malignancies and clonal hematopoiesis of indeterminate potential (CHIP) is that mutant hematopoietic stem cells (HSCs) acquire resistance to chronic inflammatory stress. In normal hematopoiesis, persistent inflammation exhausts HSC function, whereas mutant clones often withstand or even exploit this stress, conferring a selective advantage [1–4]. This principle is particularly relevant in myeloproliferative neoplasms (MPNs), where it is increasingly likely that inflammatory stressors serve as key drivers of the emergence of MPN driver clones. The sources of such inflammation may be diverse, including genetic predisposition to autoimmunity, infectious exposures, and chronic lifestyle factors.

A history of autoimmune disease has been associated with increased risk of developing MPN, supporting the idea that inherited inflammatory or immunoregulatory traits may underlie susceptibility to MPN driver mutations [5, 6]. In addition, first-degree relatives of MPN patients have a markedly elevated risk (approximately 5- to 7-fold higher) of developing MPN themselves, underscoring the contribution of germline predisposition and potentially shared immune-inflammatory backgrounds [7]. Genome-wide association studies have further identified germline single-nucleotide polymorphisms (SNPs) linked to immune regulation, such as variants at JAK2 46/1 (GGCC) and in inflammatory loci (e.g., TERT, SH2B3), which predispose to MPN development [8, 9]. Notably, loss-of-function variants in the IL6R locus, which reduce IL-6 signaling, have been shown to be protective against both MPN development and cardiovascular disease in individuals with clonal hematopoiesis, underscoring the central role of IL-6–mediated inflammation in shaping clonal dynamics [10, 11]. Our group has shown that MPN patients harbor defective interleukin-10 receptor (IL-10R) signaling, a critical negative regulator of pro-inflammatory cytokine production such as TNFα [12]. This defect was also present in an unaffected twin of an MPN patient, suggesting a heritable predisposition. Supporting this, experimental blockade of IL-10R signaling promotes the expansion of Jak2V617F mutant cells in mouse models [13], raising the possibility that impaired IL-10R signaling creates a selective niche favoring JAK2-mutant hematopoiesis.

Infectious exposures may contribute to the initiation and progression of MPN and other hematologic malignancies by creating recurrent inflammatory stress. In a large UK population-based case–control study, a prior history of cellulitis was associated with a significantly increased risk of developing MPN [14]. Similar registry-based studies in myeloid and lymphoid malignancies have shown that antecedent infections, sometimes recorded years before diagnosis, are linked to elevated incidence of AML, MDS, and lymphomas [15–17].

Lifestyle exposures clearly influence the selective pressures that shape clonal hematopoiesis and the development of MPN. Cigarette smoking, for example, has been repeatedly associated with increased risk of MPN in population-based cohorts [18]. Experimental models provide a mechanistic basis for this association, showing that cigarette smoke creates an inflammatory marrow niche that promotes the expansion of both Jak2V617F and Tet2-deficient hematopoietic stem cells [19]. Diet quality is also emerging as a relevant factor. In the UK Biobank, adherence to pro-inflammatory dietary patterns was associated with a higher prevalence of CHIP, whereas nutrient-dense, anti-inflammatory diets were linked to lower prevalence [20]. Obesity further exemplifies how metabolic inflammation accelerates clonal dynamics: in both murine models and human cohorts, obesity promotes expansion of CHIP clones, including those bearing JAK2 mutations, and exacerbates associated inflammatory cardiovascular phenotypes [21].

Acute, high-intensity exposures provide another window into how inflammation reshapes hematopoiesis. World Trade Center first responders have been shown to harbor a striking burden of clonal hematopoiesis, with enrichment of mutations linked to inflammatory signaling pathways [22, 23]. These findings suggest that sudden, intense inflammatory insults can accelerate the emergence or selection of mutant clones in otherwise healthy individuals.

Together, these epidemiologic, mechanistic, and exposure-related data converge on a consistent theme: an inflammatory milieu fosters the emergence and expansion of MPN driver clones. The relative rarity of JAK2V617F and other MPN driver mutations in unselected populations, compared with more common mutations such as TET2 or DNMT3A, suggests that JAK2-mutant clones may require specific inflammatory contexts or cooperating events to thrive, while epigenetic regulator mutations are able to expand in the more ubiquitous inflammatory environments of aging [24]. Importantly, phylogenetic studies indicate that the latency from acquisition of a JAK2 mutation to overt MPN can span decades, on the order of 30 years in some individuals [25, 26]. This long preclinical interval creates a substantial window for prevention. Lifestyle interventions that reduce inflammatory stress, including diet modification, weight management, and smoking cessation, represent attractive strategies to alter clonal trajectories. Individuals with JAK2-mutant CHIP or those with strong family histories of MPN may be especially well suited for early prevention trials.

### Inflammation and Symptom Burden

Symptoms remain one of the most prominent and distressing aspects of MPN, cutting across all disease subtypes. Patients with polycythemia vera often report severe pruritus, those with essential thrombocythemia experience microvascular problems such as headaches and erythromelalgia, and fatigue is almost universal in myelofibrosis, where it substantially impairs daily life [27]. The mechanisms behind these symptoms are complex, but inflammatory cytokines appear to play a central part. Interleukin-6, tumor necrosis factor α, and interleukin-1β are frequently elevated in patients with high fatigue scores and constitutional complaints [28, 29]. Cytokine profiles also carry prognostic information. High circulating interleukin-8 predicts inferior survival in myelofibrosis [28], and distinct cytokine signatures in essential thrombocythemia have been shown to align with mutational background and clinical behavior [30]. Together these observations suggest that cytokines influence not only how patients feel but also how the disease behaves, raising the possibility that cytokine profiling could serve as a useful biomarker in the clinic.

Therapy further illustrates the centrality of inflammation. Ruxolitinib produces rapid and often striking improvements in constitutional symptoms and quality of life in myelofibrosis, and it reduces pruritus in polycythemia vera [29, 31]. These benefits typically appear within the first few weeks of therapy, suggesting that the dominant effect is through dampening inflammation rather than directly suppressing the malignant clone. Symptom relief has also been observed with the selective JAK1 inhibitor itacitinib, which reduced fatigue and constitutional symptoms in myelofibrosis even though effects on splenomegaly and clonal metrics were modest [32]. Taken together, these findings make clear that inflammatory signaling is central to the symptom burden in MPN, and that interrupting these pathways can bring meaningful improvements to patients’ lives.

Looking ahead, cytokine signatures may also have value as prognostic or stratification tools. Current risk models such as DIPSS and MIPSS rely on clinical variables and mutational data, but the addition of cytokine profiling could refine prognostication by identifying patients at higher risk of aggressive disease or severe symptom burden. Integrating cytokine patterns with established scoring systems might allow for a more personalized approach, guiding both therapeutic selection and the intensity of monitoring.

### Measuring Inflammation in MPN

Quantifying inflammation in myeloproliferative neoplasms (MPN) remains difficult. Cytokine profiling has been the most common approach in clinical research, yet each trial has used different panels, assays, and sampling schedules, which limits comparability. In the ruxolitinib COMFORT trials, multiplex profiling showed on-treatment reductions in IL-6, TNF-α, and IL-1RA, and these changes paralleled improvements in symptom scores [29]. For momelotinib, early phase work demonstrated a rapid fall in IL-6 within six hours of the first dose, accompanied by reductions in pSTAT3 and hepcidin, highlighting a direct anti-inflammatory effect [33, 34]. In the pelabresib MANIFEST trial, serial cytokine analyses showed declines in NF-κB–regulated cytokines including TNF and IL-6, with the addition of pelabresib to ruxolitinib uniquely driving down IL-8, which rose in the control arm [35]. By contrast, studies of fedratinib and pacritinib noted exploratory cytokine changes or mechanistic pathway effects (such as IRAK1/TLR inhibition for pacritinib), but without standardized panels consistently reported across trials.

Outside of clinical trials, cytokine testing is technically available but not used in routine care. At present, there is no agreement on which cytokines should be measured, what thresholds define clinically relevant inflammation, or how results should change management. Until such tests are standardized and actionable, cytokine profiling cannot be recommended in general practice. More fundamentally, cytokines may not capture the full inflammatory burden of MPN, since each patient may have distinct pathways driving disease. Thus, while inflammation is central to MPN biology, we still lack a validated and generalizable measure of it in the clinic. Looking ahead, emerging biomarkers may complement cytokine assays and eventually provide more reliable ways to quantify inflammation in MPN.

### Inflammation and the Microenvironment in Myelofibrosis

Fibrosis in myelofibrosis reflects a profound disruption of the bone marrow microenvironment, largely driven by inflammatory cytokines. There is clear evidence for two-way cross talk between malignant hematopoietic cells and stromal elements: cytokines released by the clone activate fibroblasts, endothelial cells, and mesenchymal stromal cells, which in turn generate mediators that sustain and amplify the malignant population. Whether fibrosis is a causal driver of disease or primarily a downstream effect remains uncertain, as fibrosis grade does not consistently correlate with clinical outcomes [36].

Multiple studies have identified key mediators of this process. TGF-β and PDGF signaling are central to fibroblast activation and extracellular matrix deposition [37, 38]. Megakaryocyte-derived inflammatory mediators such as CXCL4 have been shown to promote fibroblast activation and correlate with disease progression [39, 40]. Alarmins of the S100 family, particularly S100A8 and S100A9, are elevated in the plasma and marrow of patients with MF, correlate with systemic inflammatory markers, and distinguish early from advanced fibrosis [41–43]. These proteins amplify inflammation through TLR4 and RAGE signaling, creating a feed-forward loop that drives stromal activation, while preclinical inhibition of this axis with compounds such as tasquinimod reduces fibrosis and myeloproliferation [44]. More recent single-cell and spatial transcriptomic analyses have confirmed that inflammatory cytokines including TGF-β, FGF, and TNF-α are deregulated across both hematopoietic and stromal compartments, reinforcing the concept that the inflammatory milieu underlies fibrotic remodeling [45]. Fibrosis is thus an integral part of MF, but precisely how it influences disease course - whether as a driver, a byproduct, or both - remains unclear.

### Targeting Inflammation as a Therapeutic Approach

As inflammation is central to MPN pathogenesis it is only fitting that many of the therapies used affect inflammation. JAK inhibitors are a perfect example, although the driver mutations activate JAK/STAT signaling and JAK inhibitors block this abnormal signaling the blockade of JAK inhibitors also reduce inflammation in general. Case in point, JAK inhibitors are also widely used for autoimmune diseases. Aspirin, widely used for MPN for a long time, although primarily used for its thrombotic risk reduction also has anti-inflammatory properties and these properties may have unrecognized benefits in MPN beyond thrombotic risk reduction. A medication which at face value is not an anti-inflammatory but instead is a JAK/STAT signaling activator an immune stimulant is interferon-alpha, and it is ironic that this is the only drug that can lead to molecular remissions in MPN. JAK inhibitor combinations are emerging as the next wave of treatments for MPN. The JAK inhibitor add ons may target inflammation in a different way than JAK inhibitors, for example BET inhibitors such as pelabresib, impact cytokine production likely via reduction in NFKB. So, when rationally designing JAK inhibitor combos it is good to keep an eye on how they impact inflammation.

As inflammation is central to MPN pathogenesis, it is not surprising that many of the therapies used in MPN also modulate inflammatory pathways. JAK inhibitors are a prime example. Although the primary objective of their development in MPN was to counteract constitutive JAK/STAT signaling driven by MPN-associated mutations, JAK inhibition also broadly suppresses inflammatory cytokine production. Indeed, JAK inhibitors are widely used for autoimmune and autoinflammatory diseases, underscoring their potent anti-inflammatory activity. Aspirin, a mainstay of MPN management primarily for thrombotic risk reduction, also possesses anti-inflammatory properties that may confer additional, underappreciated benefits in this disease context. Conversely, interferon-α, which paradoxically acts as an immune stimulant and JAK/STAT pathway activator, is the only therapy capable of inducing molecular remissions in MPN, highlighting the complex relationship between immune activation and disease control. Emerging JAK inhibitor combination strategies represent the next wave of MPN therapy. Many of these combinations pair JAK inhibition with agents that modulate inflammation through complementary mechanisms, for example, BET inhibitors such as pelabresib reduce cytokine production by attenuating NF-κB–mediated transcription. Thus, when designing rational JAK inhibitor–based combinations, it is essential to consider how each agent impacts the inflammatory milieu that underlies MPN biology.

### Differences in Spectrum of Targets for JAK Inhibitors in Myelofibrosis: Potential Impact of Additional Targets

All four JAK inhibitors currently FDA approved for myelofibrosis (ruxolitinib, fedratinib, pacritinib, and momelotinib) share JAK2 as their primary therapeutic target [46, 47]. However, these agents differ significantly in their selectivity profiles for other kinases, particularly JAK1, which has important implications for both efficacy and tolerability. Ruxolitinib and momelotinib inhibit both JAK1 and JAK2 with comparable potency, whereas fedratinib and pacritinib are relatively selective for JAK2 with minimal JAK1 inhibition [46, 48, 49].

JAK1 inhibition confers both potential benefits and drawbacks. On the beneficial side, JAK1 is a critical mediator of inflammatory cytokine signaling, including IL-6, and its inhibition contributes to the robust suppression of constitutional symptoms observed with JAK1/2 inhibitors [46, 47]. On the other hand, JAK1 inhibition may contribute to myelosuppression and immunosuppression, as JAK1 plays essential roles in hematopoietic cytokine signaling and lymphocyte function [50]. The relatively JAK2-selective agents fedratinib and pacritinib may thus offer advantages in cytopenic patients where preservation of marrow function is a priority.

Beyond JAK selectivity, individual JAK inhibitors possess unique off-target activities that may contribute to their clinical profiles. Pacritinib is a potent inhibitor of interleukin-1 receptor-associated kinase 1 (IRAK1), a key mediator of toll-like receptor (TLR) and NF-κB signaling pathways [51]. IRAK1 inhibition provides an additional mechanism by which pacritinib may suppress inflammatory cytokine production independent of JAK2 blockade. In murine models of myelofibrosis driven by inflammation (miR-146a knockout mice), dual JAK2/IRAK1 inhibition with pacritinib prevented splenomegaly, reticulin fibrosis, and osteosclerosis while attenuating the proinflammatory environment and reducing cytokines such as CXCL1 and TNF-α [52]. This dual targeting of JAK2 and IRAK1 may be particularly advantageous in myelofibrosis given the central role of NF-κB-mediated inflammation in disease pathogenesis [53].

Fedratinib exerts off-target inhibitory activity against bromodomain-containing protein 4 (BRD4), a member of the bromodomain and extraterminal domain (BET) protein family [47]. BET proteins play fundamental roles in cellular proliferation and proinflammatory signaling by regulating transcription of target genes including NF-κB-mediated cytokine production [54]. Preclinical studies have demonstrated that combined JAK/BET inhibition leads to marked reduction in serum inflammatory cytokines and reversal of bone marrow fibrosis in murine models [53]. Although whether the modest BRD4 inhibition achieved by fedratinib at clinical doses contributes meaningfully to its therapeutic effects remains uncertain, the success of dedicated BET inhibitors such as pelabresib in combination with ruxolitinib validates the concept of dual pathway targeting [53].

Both momelotinib and pacritinib also inhibit activin A receptor type 1 (ACVR1), which mediates SMAD2/3 signaling and contributes to upregulation of hepcidin production [48, 55]. Hepcidin is the master regulator of iron homeostasis, and elevated hepcidin in myelofibrosis contributes to iron-restricted erythropoiesis and anemia. Inhibition of ACVR1 suppresses hepcidin production, increases iron availability, and improves erythropoiesis [56]. This mechanism underlies the unique anemia benefits observed with momelotinib and pacritinib, with recent data demonstrating that pacritinib is the most potent ACVR1 inhibitor among the approved JAK inhibitors [55]. In summary, the four approved JAK inhibitors for myelofibrosis, while sharing JAK2 as a common target, possess distinct profiles of additional kinase activities that contribute to their differential clinical effects on inflammation, anemia, and cytopenias.

## Conclusion

Inflammation is intricately interwoven into every aspect of myeloproliferative neoplasm (MPN) biology, from clonal initiation and disease propagation to symptom burden and fibrotic remodeling. The distinction between the malignant clone and its inflammatory microenvironment is increasingly indistinct; inflammation is not merely a byproduct of the disease but a co-driver of its evolution. Although recent advances have begun to untangle the complex interplay between neoplastic and inflammatory pathways in MPN, the underlying network remains far from resolved. Viewing MPN through the lens of inflammation reframes it not simply as a neoplasm, but as a maladaptive immune–stromal ecosystem. Therapies that restore equilibrium within this system, by dampening pathologic inflammation while preserving immune competence, may ultimately hold the key to durable disease control and, perhaps, prevention.

## Data Availability

No datasets were generated or analysed during the current study.
